# Process-Induced Molecular-Level Protein–Carbohydrate–Polyphenol Interactions in Milk–Tea Blends: A Review

**DOI:** 10.3390/foods13162489

**Published:** 2024-08-08

**Authors:** Dilema Wijegunawardhana, Isuru Wijesekara, Rumesh Liyanage, Tuyen Truong, Mayumi Silva, Jayani Chandrapala

**Affiliations:** 1 School of Science, STEM College, RMIT University, Bundoora, VIC 3083, Australia; S3934930@student.rmit.edu.au (D.W.); tuyen.truong@rmit.edu.au (T.T.); mayumi.silva@rmit.edu.au (M.S.); 2 Department of Biosystems Technology, Faculty of Technology, University of Sri Jayewardenepura, Dampe-Pitipana Road, Homagama 10200, Sri Lanka; rumeshliyanage@sjp.ac.lk; 3 Department of Food Science and Technology, Faculty of Applied Sciences, University of Sri Jayewardenepura, Gangodawila, Nugegoda 10250, Sri Lanka; isuruw@sci.sjp.ac.lk; 4 School of Science, Engineering & Technology, RMIT University, Ho Chi Minh City 700000, Vietnam

**Keywords:** milk–tea, protein, carbohydrate, polyphenol, pasteurization, concentration, spray drying

## Abstract

The rapid increase in the production of powdered milk–tea blends is driven by a growing awareness of the presence of highly nutritious bioactive compounds and consumer demand for convenient beverages. However, the lack of literature on the impact of heat-induced component interactions during processing hinders the production of high-quality milk–tea powders. The production process of milk–tea powder blends includes the key steps of pasteurization, evaporation, and spray drying. Controlling heat-induced interactions, such as protein–protein, protein–carbohydrate, protein–polyphenol, carbohydrate–polyphenol, and carbohydrate–polyphenol, during pasteurization, concentration, and evaporation is essential for producing a high-quality milk–tea powder with favorable physical, structural, rheological, sensory, and nutritional qualities. Adjusting production parameters, such as the type and the composition of ingredients, processing methods, and processing conditions, is a great way to modify these interactions between components in the formulation, and thereby, provide improved properties and storage stability for the final product. Therefore, this review comprehensively discusses how molecular-level interactions among proteins, carbohydrates, and polyphenols are affected by various unit operations during the production of milk–tea powders.

## 1. Introduction

Drinking tea with milk is deeply rooted in consumers’ daily lives in many countries, such as the United Kingdom, Germany, France, Italy [1], Ireland, Canada [2,3], China [4], Japan, Australia, Taiwan [1], Pakistan, India [5], and Sri Lanka [6]. The global market for milk–tea was worth USD 2.4 billion in 2019 and was projected to reach to USD 4.3 billion by 2023, serving as evidence of its popularity [1]. The potential to modify its organoleptic and nutritional qualities is what primarily encourages this pervasive practice of drinking milk–tea [7,8,9]. The powdered form of milk–tea blends creates new market opportunities around the globe due to the increase in population, urbanization, and ease of transportation [10,11,12].

In commercial-level production, milk–tea, like milk-containing formulas, are further combined with various additives, such as maltodextrin, fructans, galacto-oligosaccharides, lactose, and glucose polymers, to optimize the physiochemical, nutritional, and sensorial properties [13,14]. Maltodextrin is frequently used as a safe additive accepted by the Federal Drug Administration (FDA) [15] to enhance the particle size, bulk density, solubility [16], viscosity, volume, oral sensation, and stability of milk-containing formulas [17]. This high compositional diversity promotes various interactions within the formulation and can cause changes in the expected compositional consistency, physical, sensorial, and functional properties of the ultimate product. Some of these changes can be easily identified by consumers; so, it is always important to carefully control and adjust the internal composition and processing parameters to ensure the desired qualities of the product [18,19,20]. Modifying the internal composition and processing conditions induces structural changes in inside molecules, thereby redesigning the molecular grafting, conjugation, or polymerization. These reconfigured molecules could provide desirable changes to the physical and chemical properties of the product, thus influencing the expected properties of the final product [21].

Milk–tea is a complex blend of proteins, carbohydrates, and polyphenols, making it crucial to understand how these components interact during processing, as these interactions directly impact sensory attributes, nutritional value, functionality, and rehydration properties. Among these components, native proteins play a significant role in modifying these interactions, particularly when exposed to heat due to their unique structural arrangements [22,23,24]. Heat causes proteins to undergo structural rearrangements, leading to changes in inter- and intra-molecular interactions. These interactions include protein–protein, protein–carbohydrate, protein–polyphenol, and protein–carbohydrate–polyphenol conjugations [25,26,27]. Carbohydrates and phenols also interact with each other to make self-carbohydrate and carbohydrate–phenol interactions, which are not frequent as with proteins. However, despite the molecules involved, this modification could cause significant changes to the ultimate milk–tea properties and functionalities [28,29].

**Figure 1:**
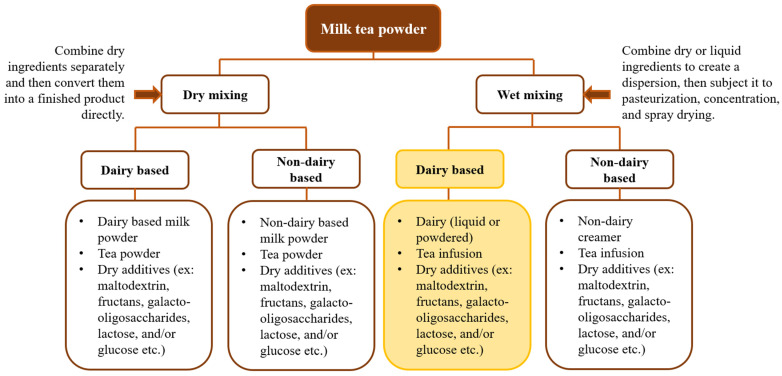
Two methods for preparing milk–tea powder: wet mixing and dry mixing. Each method involves two different types of mixtures, including dairy-based and non-dairy-based options.

Commercial milk–tea powder can be produced using two methods: dry mixing and wet mixing, as shown in Figure 1. In the dry mixing method, tea powder and milk powder are blended directly [30]. Wet mixing involves mixing a tea infusion with liquid milk, pasteurization, concentration, and drying to produce powdered milk–tea blends. This process ensures the even distribution of nutrients, reduces reliance on base ingredients for microbiological quality, and has thus been shown to be more advantageous than the dry mixing method. Therefore, this article discusses the molecular-level interaction modification associated with the wet mixing method of preparing milk–tea [31,32,33].

The impact of pasteurization, evaporation, and spray drying on the physicochemical, structural, and functional properties of powdered milk–tea blends has not been investigated in the previous or recent literature. While much research has focused on the sensory and nutritional aspects of liquid milk–tea after preparation [9,34,35,36,37,38], there is limited information on the preparation of powdered milk–tea formulations. The process of pasteurization, concentration, and spray drying in the production of powdered milk–tea can cause significant changes in the structure of its components. This could affect intermolecular and intramolecular interactions among key elements such as proteins, carbohydrates, and tea polyphenols. Therefore, it is essential to gain a thorough understanding of these interactions and structural changes that occur within major biopolymers such as proteins, carbohydrates, and tea polyphenols during the pasteurization, concentration, and spray drying stages in the wet mixing method of milk–tea powder production. This understanding is crucial for customizing the properties of the final product. Therefore, this review provides a thorough examination of how different dairy-based milk–tea ingredients interact during the wet mixing stages of processing, such as pasteurization, evaporation, and concentration, in milk–tea powder production. 

## 2. Bovine (Cow) Milk

Milk is a biological, complex, multi-phase colloidal suspension that is composed of 85–90% water, followed by lactose (3.6–5.5%), fat (2.5–6.0%), proteins with essential amino acids (2.9–5.0%), minerals (0.8–0.9%), vitamins (0.1%), and enzymes (60) [11,39,40]. The concentration of the principal and minor constituents in milk varies widely among species, individual animals, breeds, stage of lactation [41], genetics, and environmental conditions [42]. Consequently, the compositional variation in raw bovine milk could slightly affect the properties of the ultimate milk–tea mixtures.

### 2.1. Milk Protein

The two major milk proteins, caseins and whey proteins, play significant roles when exposed to heat [43,44]. The specific primary structures among caseins (α_S_- (α_S1_- and α_S2_-), β, and κ-casein) and whey (β-lactoglobulin (β-Lg), α-lactalbumin (α-La), blood serum albumin (BSA), and immunoglobulin (Ig)) encourage individual proteins to differ from each other and show distinct association behaviors with the available components during product formulation and processing. 

The availability of a random and open structure with high amounts of proline (Pro), few amounts of cysteine (Cys) residues, and hydrophobic domains with an amphiphilic nature facilitate caseins to show a high tendency of interaction among themselves and the other available proteins in milk–tea [45,46]. The Cys residues are present in α_S2_- and κ-caseins and, therefore, associate through intermolecular disulfide bonds. In contrast, the lack of cysteine or cystine residues increases the flexibility of α_S1_- and β-caseins and prevents precipitation through sulfhydryl–disulfide interchange crosslinking reactions [47,48]. Non-covalent casein interactions (such as weak hydrophobic, hydrogen, and electrostatic interactions) arise with the presence of pro and glutamine (Gln) residue-rich sequential neighbors and far sequences [49]. The native protein structures are stabilized through these forces in a mixture. However, both internal (amino acid residues in the sequence, surface charge, molecular weight, and conformational state) and external (ionic strength, pH, temperature, and pressure) factors affect the formation, extent, and stability of these interactions [50,51,52,53]. 

Compared to caseins, whey proteins consist of folded and compact molecular structures, predominantly present in the form of dimers in milk at room temperature. β-Lg, which is the major whey protein (10–15% of the total protein and half of the total whey proteins), begins to dissociate into monomers when heated at temperatures above 40 °C. This process leads to the partial unfolding of β-Lg and the disruption of its helical structures, followed by a decrease in the β-sheet content when the temperature is increased from 40 °C to 60 °C. The loss of helical structures encourages conformational changes in β-Lg (>65 °C) by exposing its free thiol, Cys 121, to form disulfide bridges and interact with other molecules. The conformational changes in β-Lg result in the formation of a disulfide bond between Cys 106 and Cys 121, replacing Cys 119 in the Cys 106–Cys 119 disulfide bond. The free thiol containing Cys 119 triggers aggregation with other proteins that have a disulfide bond, such as α-La. Consequently, these intra- and inter-molecular disulfide exchange reactions that occur between β-Lg and β-Lg or β-Lg with other disulfide-containing proteins affect the solubility and functionality (hydration, viscosity film formation, and gelling) of proteins [54,55,56,57,58]. However, the heat resistance of α-La, which comes from the four disulfide linkages, with no free sulfhydryl groups, makes β-Lg the highest contributor to these inter- and intra-molecular interactions and the irreversible heat denaturation, aggregation, and gelling properties of proteins in milk [59,60,61]. Apart from thiol groups, hydrophobic pockets in whey proteins are also involved in the aggregation and/or polymerization of molecules through both covalent and non-covalent intermolecular linkages [58]. In addition to temperature, the pH of the medium largely affects these interactions. At ambient temperature and neutral pH, whey proteins are present in the form of a dimer, while at and acidic pH, dimers dissociate into monomers [62]. For instance, β-Lg self-assembles to form irreversible aggregates referred to as amyloid fibrils when heated at low pH conditions [63].

### 2.2. Carbohydrates in Milk

**Figure 2:**
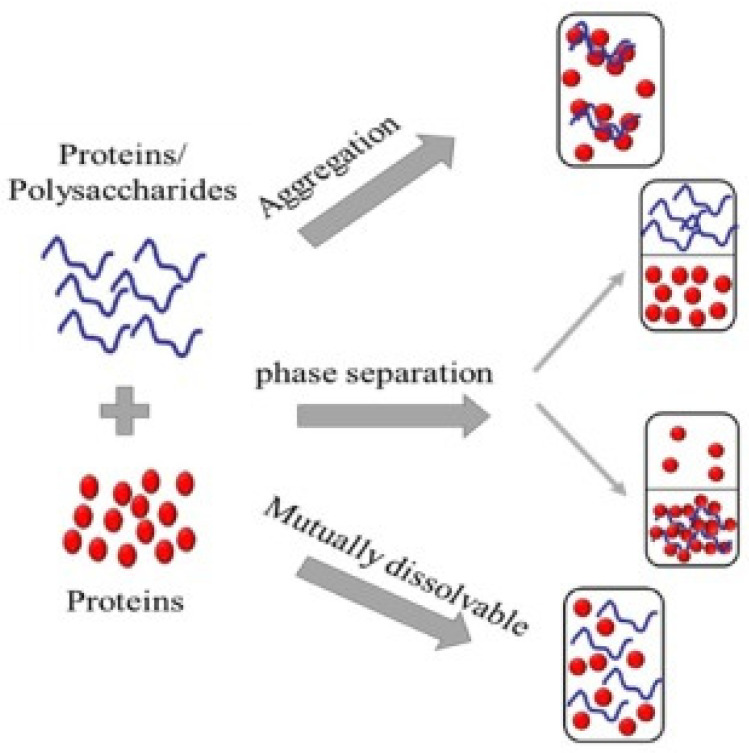
Various ways in which proteins and polysaccharides can interact and form structural arrangements, including aggregation, phase separation, and mutual dissolution, when mixed together [26]. Copyright 2023, Elsevier.

Milk carbohydrates primarily refer to lactose, which constitutes approximately 98% of the total carbohydrates in milk. The remaining carbohydrates include minor amounts of glucose and galactose, which are not bound within the lactose molecule and exist in their free, unbound forms [64,65]. The interactions with medium components, such as proteins and bioactive components, are supported by the amphiphilic nature of the carbohydrate molecule either through association or repulsion interactions and thereby result in complex or immiscibility of the two biopolymers. Consequently, the immiscibility of biopolymers leads to phase separation in the mixture, as shown in Figure 2, based on the nature of the components, composition of the formulation, and the environmental conditions [66,67,68]. These protein–carbohydrate attractive interactions occur directly, as well as indirectly, and are dominated by covalent, ionic, hydrogen, and van der Waals interactions and physical entanglements [69,70]. However, the low molecular weight of lactose provides a better accessibility for proteins in contrast to maltodextrin to form protein–carbohydrate interactions [71]. Apart from protein–carbohydrate interactions, at higher carbohydrate concentrations, preferential sugar–sugar interactions can lead to phase separation into carbohydrate-rich and protein-rich phases [29]. This occurs when proteins and carbohydrates have the same charge, causing them to be separated by electrostatic repulsion and resulting in their presence in two distinct phases. This separation can lead to the formation of an insoluble complex, which subsequently condenses and precipitates [26]. The use of maltodextrin, an effective emulsifier, enhances the dispersion of proteins and carbohydrates, thereby mitigating phase separation and improving overall stability [72]. Additionally, maintaining an optimal protein-to-carbohydrate ratio stabilizes the formulation by creating a homogeneous matrix, which further prevents phase separation. This stability enhances texture and contributes to improved formulation stability. These protein–carbohydrate interactions and re-arrangements in milk–tea blends affect water-holding capacity, gelling, film forming, viscosity and rheological behavior, crystal growth inhibition, and sensory profiles [58]. 

## 3. Milk–Tea Blends

Tea contains polyphenol flavonoids (250–350 g/kg) along with small amounts of carbohydrates, proteins, vitamins, and minerals [73]. The major phenolic class of thearubigins (54.8%), followed by gallic acid derivatives (8.9%), flavonols (8.6%), theaflavins (7.3%), hydroxycinnamates (7%), and flavan-3-ols (3.3%), are reported to be available in black tea infusions [74]. When milk is combined with tea, it increases the complexity of the mixture, ultimately affecting the perceived palatability [75,76,77]. Additionally, combining tea as a secondary additive promises higher health-promoting effects on milk [14], such as chemo-preventive activity against various cancers [78,79], antioxidant effects against oxidative damage [80], a lowering effect on coronary heart disease, and protection against dental caries and bone loss [81]. 

The wet mixing method of milk–tea powder production involves pasteurization, concentration, and drying. Pasteurization is the process used to make milk-containing formulas to ensure microbial safety [82]. The dairy industry commonly uses two pasteurization methods: low-temperature long-time (LTLT) at 63 °C for 30 min and high-temperature short-time (HTST) at 72 °C for 15 s [83]. Concentration is an intermediate step in the production process. Concentration partially removes water and thereby increases the nutrient density of protein, fat, sugars, and minerals in a unit volume compared to the initial liquid formulation [70,84,85,86]. Concentration through evaporation is an energy-intensive process that removes water under partial vacuum and elevated temperatures (45–75 °C) [84]. Dehydration completely removes the water from the concentrated formula. This process involves replacing water molecules with other components, such as proteins, carbohydrates, and polyphenols [87]. The most commonly used method for dehydrating milk–tea is spray drying, as it provides a high yield of the final powder [88,89]. 

## 4. Interactions in Milk–Tea Blends: During Pasteurization, Concentration, and Spray Drying

**Figure 3:**
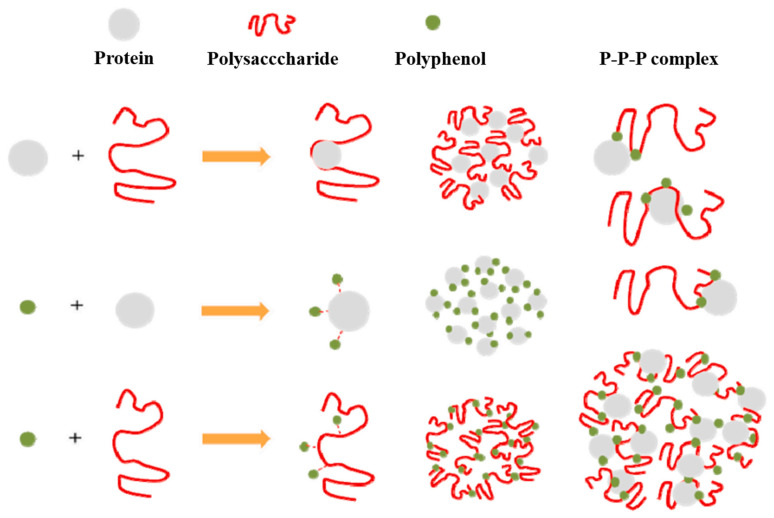
The mechanism of protein–polysaccharide–polyphenol interactions in foods includes protein–polysaccharide, protein–polyphenol, and protein–polysaccharide–polyphenol interactions [94]. Copyright 2023, Elsevier.

Interactions between proteins (whey and casein) and carbohydrates (including lactose and maltodextrin) in milk and polyphenols in tea occur both covalently and non-covalently [90]. Covalent interactions are formed through an equally shared pair of electrons by two atoms in the same type of molecule (protein–protein) or with another type of molecule (protein–phenol) via C–C, C–O, C–N, or C–S linkages [91,92]. Non-covalent binding occurs through hydrophobic, van der Waals, hydrogen bridge, and ionic interactions and is weaker in strength compared to covalent bonding, due to the absence of electron pair-sharing action and the nature of reversibility [93]. These interactions in milk–tea mixtures are affected by the phase transition directed by heating. The ways of associations between various components, such as proteins, carbohydrates, and polyphenols, are often known as the co-action of macro–macro molecules (protein–polysaccharide), macro–micro molecules (protein and/or polysaccharide with polyphenols), and micro–micro (polyphenol–polyphenol) molecules, as shown in Figure 3.

### 4.1. Protein–Protein Interactions

Casein micelles are remarkably stable at high temperatures and disassociate at temperatures above 100 °C [95]. Whey proteins, such as BSA and Ig, start denaturing at around 65 °C and irreversibly denature at approximately 70 °C, with thiol exposure occurring at about 72 °C [96,97]. The major whey proteins, such as β-lg and α-La, undergo their significant denaturation only at temperatures in the range of 70–75 °C [98]. This denaturation induced by pasteurization promotes the binding of whey to whey and whey to casein in liquid milk-containing formulas, such as milk–tea. 

Whey–whey interactions can occur directly or indirectly during pasteurization. On the one hand, denaturation causes conformational changes in the structure of α-La and β-Lg (~75 °C), promoting their direct interaction through hydrophobic interactions. This interaction can occur among β-Lg-β-Lg, β-Lg-α-La, and α-La-α-La, resulting in the formation of soluble aggregates [99]. Whey–casein complexes connect with other denatured whey proteins that have free thiol groups, such as BSA, through S-S/S-H interchange reactions [100]. As a result, whey proteins polymerize and form an invisible gel network, increasing the viscosity of the pasteurized milk–tea. 

Heat-induced conformational changes in β-Lg lead to the formation of disulfide bonds with casein proteins that contain cysteine or disulfide bridges, such as α_S2_-casein, and κ-casein [101,102]. β-Lg–κ-casein complexes are the primary and abundant complexes (at pH~7.0) that form between casein and whey. β-Lg binds to free and surface-active κ-casein at temperatures of ~80 °C or higher, leading to the association of mutual casein micelles and the formation of minor-level aggregates. In addition to β-Lg, α-La is also involved in forming complexes with casein micelles [103,104,105]. Mutual casein interaction is facilitated by hydrophobic interactions involving specific amino acid residues, such as Ile, Leu, Phe, Trp, Met, Tyr, and Val [53]. 

Interactions between casein–casein, whey–whey, and whey–casein ultimately cause protein particles to aggregate, resulting in increased bulk viscosity in the pasteurized formula [106,107,108,109,110]. However, Andersson et al. (2021) [111] found that highly denatured and aggregated proteins are undesirable in the final powder due to low solubility. Therefore, it is important to control protein denaturation and aggregation during pasteurization by adjusting the processing conditions and initial blend composition.

**Figure 4:**
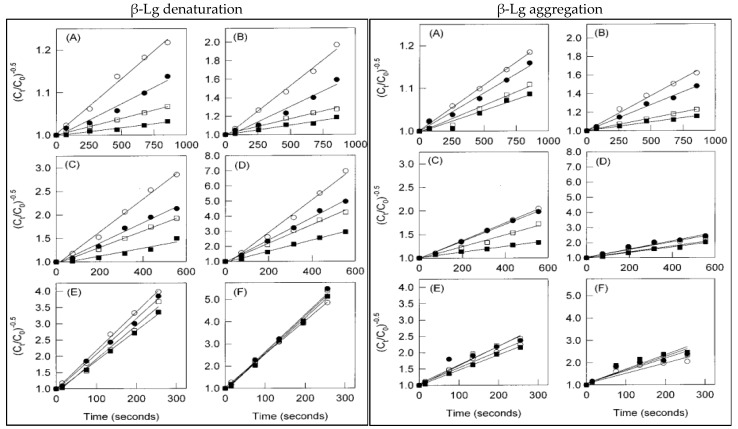
Denaturation and disulfide aggregation of β-Lg as a reaction order of 1.5: (**A**) 75 °C; (**B**) 80 °C; (**C**) 85 °C; (**D**) 90 °C; (**E**) 95 °C; and (**F**) 100 °C (◯, 9.6% TS; ●, 19.2% TS; □, 28.8% TS; ■, 38.4% TS (*C*_0_—initial native protein concentration, *C_t_*—native protein concentration at time [112].

During concentration, whey protein plays a more significant role in forming interactions compared to casein, due to its heat-sensitive structure. However, there have been very few studies focusing on whey protein denaturation and interaction formation in milk-containing mixtures with higher solid concentrations. Anema (2000) [112] conducted a study to examine the impact of milk concentration on the irreversible thermal denaturation and disulfide aggregation of β-Lg. The total solids (TS) contents of reconstituted skim milk samples were maintained at a range from 9.6% to 38.4%. These concentration levels were achieved by heating the samples at a wide temperature range of 75–100 °C for approximately 15 min. The study observed a strong retardation in the denaturation and disulfide aggregation of β-Lg with the increase in the TS content at 80 °C, as shown in Figure 4. However, it was found that increasing the TS content (up to 38.4%) at 95 °C was less effective in slowing down the denaturation and disulfide aggregation of β-Lg. Moreover, the study compared the level of denaturation and aggregation of β-Lg. The average level of β-Lg denaturation was 15% greater than the level of β-Lg disulfide aggregation. This higher aggregation of β-Lg was due to the involvement of a minor degree of non-covalent interactions (hydrophobic) in addition to disulfide aggregation. The thiol–disulfide exchange reaction of denatured β-Lg with κ-casein and α_s2_-casein was also predicted to result in a higher level of β-Lg disulfide aggregation.

During spray drying, protein–protein aggregation occurs through various types of interactions. These interactions can be either covalent, such as inter- and intra-molecular disulfide bonds, or non-covalent, including hydrophobic, hydrogen, ionic, and other weak interactions. These solid-state protein–protein interactions are greatly influenced by whey protein denaturation, which is controlled by the inlet temperature of the spray dryer [113]. Typically, milk-containing formulas are subjected to inlet and outlet temperatures in the ranges of 180–200 °C and 80–100 °C, respectively [114]. However, a study conducted by Rogers et al. (2012) [115] contradicts this and suggests that using an inlet temperature in the range of 150–180 °C can lead to the partial insolubility of milk powders, indicating heat damage to the proteins. Temperatures above 180 °C were found to make the powders completely insoluble due to increased protein damage and subsequent protein–protein aggregation. The study also discovered that using a lower inlet temperature of 150 °C reduced the wetting time of the powder to 2 ± 1 min, compared to other inlet temperatures of 90, 111, 135, and 165 °C. This is because, at lower temperatures, proteins become the predominant component on the surface of spray-dried dairy particles. Consequently, there is less protein damage on the surface, leading to a reduction in protein–protein interactions. This, in turn, enhances the wettability and solubility of the particles [116].

However, whey protein has a lower impact on the formation of protein–protein interactions during drying compared to casein. This is due to the limited availability of whey protein. In contrast, casein forms interactions with casein through hydrophobic interactions, resulting in significant agglomeration and insolubility [116,117,118]. One potential solution to mitigate this insolubility is to introduce an intermolecular space between casein molecules, thereby reducing their self-association [119]. Another approach involves incorporating excipient substances, such as lactose or maltodextrin, which can also aid in reducing protein aggregation during the drying process [120]. Additionally, maintaining low outlet temperatures in the range of 60–80 °C also prevents the over-denaturation of whey proteins and reduces protein insolubility [121]. Reducing protein–protein aggregation ultimately enhances the processing and storage capabilities of milk–tea powder [120]. Therefore, independently selecting the inlet and outlet temperatures reduces the extent of protein–protein interactions and optimizes the wettability and solubility of the drying powder.

### 4.2. Protein–Carbohydrate Interactions

Protein–carbohydrate associations in foods can occur in different ways, including physical bonding such as van der Waals, electrostatic, hydrophobic, hydrogen, or chemical, as in the case of the Maillard reaction [122]. The Maillard reaction is the most common chemical reaction that can occur during the pasteurization of milk–tea due to the availability of lactose and free amino acid side chains of proteins. Caseins frequently contribute the Maillard reaction due to their high concentration (80% from total protein), compact structure, and the presence of a high amount of lysine (Lys) residues compared to whey proteins. Among other caseins, κ-casein shows the highest reactivity for the Maillard reaction due to the higher molecular weight, relatively extended structure, and flexibility, which make its Lys residues more accessible for interactions with reducing sugars. Due to their increased reactivity, Lys is the prime contributor to the Maillard reaction. Whey (β-Lg) also conjugates with lactose upon thermal applications by means of the Maillard reaction. However, whey protein has fewer reactive sites available for the Maillard reaction due to reduced Lys concentration. Furthermore, the denaturation and aggregation of whey proteins caused by heat reduces their reactivity in the Maillard reaction, as there are fewer lysine residues present [123,124,125,126].

**Figure 5:**
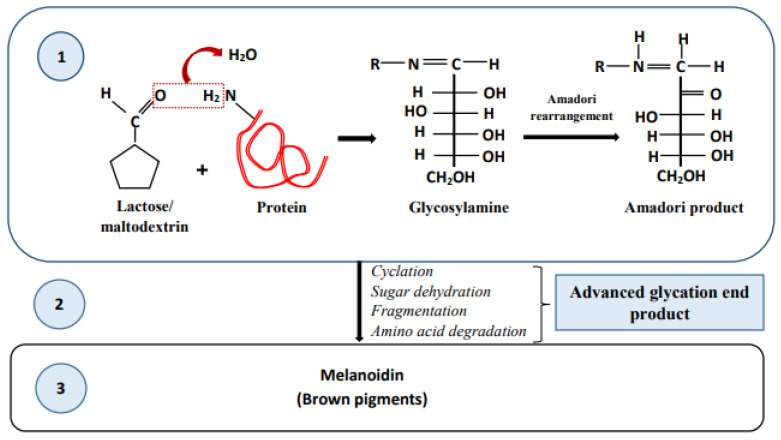
Maillard reaction in milk–tea. (1) In the early stage of heating, carbohydrates (such as lactose and/or maltodextrin) interact with proteins, initiating the Maillard reaction in milk–tea. A glycosylation reaction occurs with covalent bonding between the carbonyl group of carbohydrates and the amine group of proteins, forming an Amadori product through Amadori rearrangement. (2) Cyclation, sugar dehydration, fragmentation, and amino acid degradation reactions occur, forming advanced glycation end products. (3) In the final stage of the Maillard reaction, brown pigments are formed.

As depicted in Figure 5, the Maillard reaction involves a series of chemical reactions that occur when milk–tea is heated. Briefly, in the early stages of the Maillard reaction, glycosylation reaction occurs with covalent bonding between the carbonyl group of reducing sugars and the amine group of proteins, releasing one water molecule to form glycosylamine. This glycosylamine undergoes an irreversible rearrangement while forming the Amadori product, 1-amino-1-deoxyketose. Therefore, the nutritional value of the milk–tea is decreased at the beginning stage of the reaction due to the loss of a significant amount of amino acids [126,127,128,129]. During the intermediate stage, cyclation, sugar dehydration, fragmentation, and amino acid degradation reactions result in the sensory property changes in the product with the formation of pigment (buff-yellow color) and the generation of volatile compounds. Red/dark brown polymer (melanoidins) formation occurs in the formulation during the final stage of the Maillard reaction, negatively affecting the sensory properties even further [130,131,132]. Consequently, this reaction leads to the formation of cross-links between protein chains in the product, reducing its digestibility [133,134]. Furthermore, the Maillard reaction produces anti-oxidative compounds, such as Amadori products and melanoidins, through glucose-Lys and glucose-Gly (Glycine) interactions, and thereby, reduces the oxidative damage, which in turn results in extending the shelf life of the product [135,136]. However, the Maillard reaction in the formulation is affected by the initial pH, water activity, water content, heating method, and the type of carbohydrates involved, as described in Table 1 [137].

Maltodextrin–protein (maltodextrin–casein and maltodextrin–whey) conjugates that result from the Maillard reaction exhibit excellent protein solubility and emulsifying capabilities that are superior to those of commercial emulsifiers. The increased polarity of proteins when they are glycosylated with maltodextrin, the addition of hydrophilic saccharide groups and their steric hindrance, which reduces protein–protein interactions, all contribute to this enhanced protein solubility [144,145,146,147]. The superior emulsification ability of protein–maltodextrin conjugates in the formulation is due to two factors. First, the surface-active protein forms a stabilizing layer at the oil–water interface, preventing droplets from re-coalescing after emulsification. Second, the polysaccharide contributes to colloidal stability by thickening the mixture, resulting from the spontaneous structuring of maltodextrin in the formulation [148,149]. Consequently, the formation of protein–maltodextrin conjugates during pasteurization is beneficial for improving the emulsification ability of milk–tea formulas. However, there is a lack of recent research on the complexation of protein–lactose in milk systems during pasteurization.

Water evaporation, high lactose concentration, and reduced water activity promote the conjugation of lactose and glucose with whey and casein through the Maillard reaction [70,71,150]. This reaction leads to the production of advanced Maillard products and formic acid, causing the isomerization and degradation of lactose and a decrease in the pH of concentrated milk. Consequently, the heat stability of concentrated milk is reduced due to a decrease in the net negative charge of the casein micelles [151].

During drying, water replacement is the sole theory that explains the specific associations between proteins, such as casein and whey, and carbohydrates, such as lactose and maltodextrin. Proteins and water initially used to have hydrogen bonds; however, upon drying, the hydroxyl groups of the carbohydrate can create new hydrogen bonds with the protein. The increased surface area of the unfolded (denatured) state of the protein, compared to the folded (native) state, promotes this binding more and more. This substitution of hydrogen bonds preserves the protein structure and thereby minimizes dehydration-induced protein damage [152,153]. As a result, it ensures the thermal stability of proteins in milk–tea powder [29,154].

The amorphous structure of sugar plays a significant role in the formation of protein–carbohydrate interactions and in stabilizing the protein structure during drying. This protective mechanism of the amorphous sugar structure involves two hypotheses. The first hypothesis suggests that the sugar forms a glassy matrix that restricts the mobility and reactivity of proteins. The second hypothesis proposes that this matrix inhibits harmful intermolecular pathways by diluting the protein in the solid state and limiting intermolecular protein–protein interactions [155,156]. Initially, the glassy saccharide matrix ensures protein stability, which is then further improved by interactions between proteins and carbohydrates. Lactose is more effective than maltodextrin in stabilizing proteins during drying. This is because lactose has a smaller size and higher molecular flexibility, which enhances the miscibility between the protein and saccharides. Additionally, these tiny disaccharides have closer interactions (hydrogen bonds) with the protein surface than larger saccharides due to their lesser steric hindrances. In contrast, larger saccharides are unable to establish an intimate contact with the protein surface, potentially leading to cavities and protein instability [152].

Protein and carbohydrate concentrations also affect protein–carbohydrate interactions during drying. A low sugar concentration promotes protein stabilization through preferred protein–carbohydrate hydrogen bonding. However, increasing sugar molecules reduces the number of effective protein–carbohydrate contacts, due to an increase in carbohydrate–carbohydrate interactions, leading to less protected proteins during drying [29].

The Maillard reaction occurs once again during the process of spray drying milk–tea. The dry-state Maillard reaction involves an equal number of amino acids (Lys) and lactose. Arginine (Arg) and histidine (His) amino acids may also be involved secondarily in reacting with lactose. Lys residues show a higher reactivity towards the Maillard reaction, similar to liquid systems [157]. However, the kinetics of the Maillard reaction during spray drying differ from those of liquid systems due to the low moisture content of the particles and depend on factors, such as feed composition, pH, water, wall deposition, and outlet temperature. The Maillard reaction rate generally increases with the increase in the pH (>7), because at a low pH, only a few amino groups are available for the Maillard reaction. A lower pH (~7) is beneficial for the reaction rate in the system containing lactose because it promotes the conversion of disaccharides into monosaccharides. The Maillard reaction is maximum at a 50% moisture level, because high water activity causes a low reaction rate, while low water activity slows the reaction rate due to the lower mobility of reactants. Longer particle deposition (1–10 min) at the spray dryer wall results in a greater extent of the Maillard reaction as the Maillard reaction is time-dependent [158,159,160]. The outlet temperature exerts an extensive effect on the Maillard reaction. The extensive Maillard reaction can be seen at the inlet and outlet temperatures of 180 °C and 90 °C, respectively [150]. However, the Maillard reaction in the dry state has not been extensively studied in the literature.

### 4.3. Protein–Polyphenol Interactions

**Figure 6:**
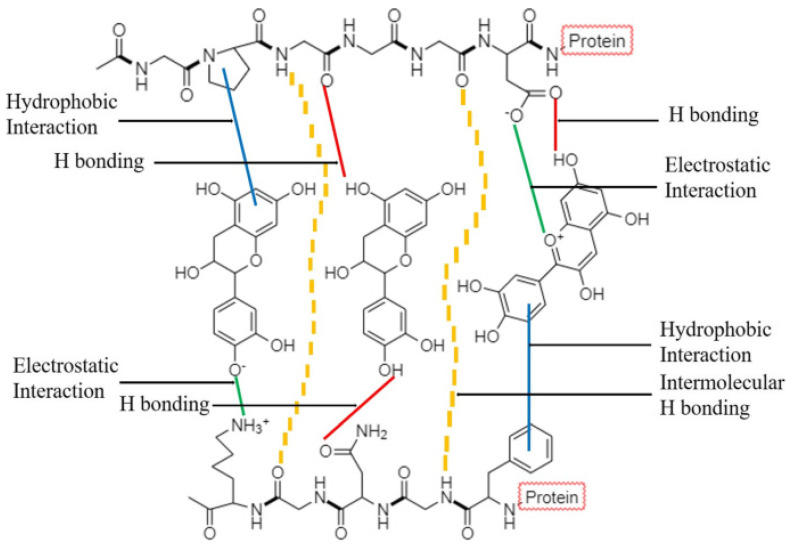
Polyphenols form major non–covalent interactions with protein molecules. Electrostatic interactions are represented by green lines, hydrogen bonding (H bonding) by red lines, and hydrophobic interactions by blue lines [170].

The strong protein–polyphenol interactions in liquid systems result from proteins unfolding during heating [161,162,163]. Two basic mechanisms are suggested for protein–phenolic associations. The first mechanism is the complexation of protein–phenolic compounds occurring either through irreversible covalent or reversible non-covalent binding, as shown in Figure 6 [164]. These associations occur when the formulation is heated to high temperatures (≥ 80 °C) [106,165,166]. Covalent interactions occur through C-N and C-S bonds between protein and phenol molecules [92]. Non-covalent interactions involve hydrogen bonding, hydrophobic interactions, and ionic interactions. Hydrogen bonding between protein and phenols occurs between phenolic hydroxyl and peptide carbonyl groups [167,168,169]. Hydrophobic interactions between protein and phenols occur between hydrophobic amino acid residues (leucine (Leu) and Gly) and the non-polar aromatic polyphenol ring. Additionally, the charged side of proteins promotes ionic interactions with polyphenols. Positively charged amino acid (Lys) residues and the negatively charged hydroxyl group of polyphenols are largely involved in ionic binding [92]. However, both covalent and non-covalent protein–phenol interactions result in protein precipitation either through multisite (a single protein binds with several phenols) or multidentate (a single phenol binds with several protein sites or molecules) interactions.

The second mechanism involves the cross-linking of polypeptide chains through the ability of phenols to create bridges between protein molecules. This cross-linking process is dependent on the concentration of protein and phenols in milk–tea. When there are more proteins than phenols, each polyphenol connects two protein molecules, forming protein dimers and small protein aggregates. On the other hand, when the phenol level increases, protein binding sites become filled with phenols, leading to the formation of a hydrophobic monolayer around the proteins and aiding in precipitation. Haze formation occurs in both scenarios, with an increase in the phenol concentration, leading to an increase in haze. This unique cross-linking mechanism ultimately influences the nutritional profile of black tea [164]. However, there are various tea polyphenols, and different proteins, such as casein and whey, bind to them in different ways, affecting the final formulation of milk–tea.

**Figure 7:**
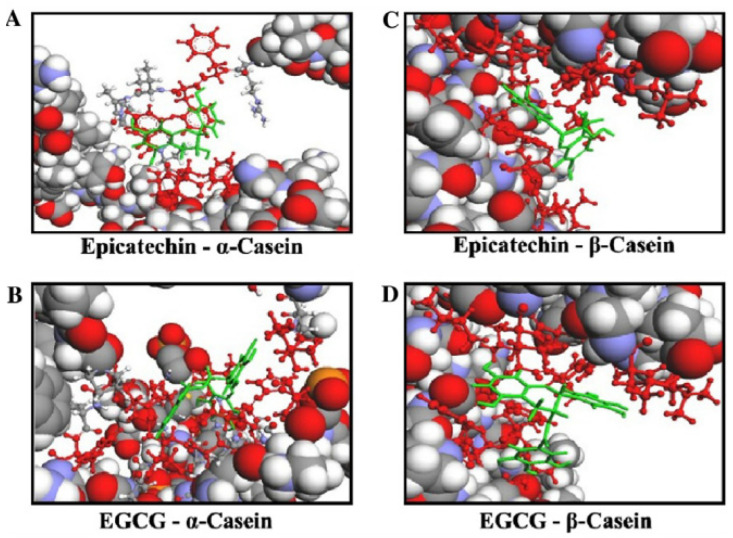
Molecular docking of catechin–casein complexes. Interested protein residues and catechin appear in red and green colors, respectively. (**A**) Epicatechin associated to α-casein; (**B**) EGCG associated to α-casein; (**C**) epicatechin associated to β-casein; and (**D**) EGCG associated to β-casein [161]. Copyright 2023, Elsevier.

Epigallocatechin gallate (EGCG) is one of the main polyphenol compounds present in tea infusions [171]. EGCG interacts with a wide range of protein varieties, especially with Pro-rich and non-globular extended proteins, such as β-casein. β-casein wraps and tightly packs itself around EGCG during heating (Figure 7) and stabilizes the casein structure. EGCG shows higher masking and combination ability (4%) compared to other tea phenolic compounds, such as catechin (0.7%), epicatechin (1.7%), and epicatechin gallate (2.9%) with β-casein. This EGCG-β-casein binding involves the hydrogen binding sites of Pro 105, Pro 168, Lys 43, Lys 44, Leu 134, Ile 222, and Tyr 208; hydrophobic binding sites of Leu 180 and Lys 43; and van der Waals binding sites of Glu 136, Glu 106, Pro 165, Pro 167, Ile 45, Ile 223, Val 212, Phe 220, Thr 135, Leu 166, and Asn 42. However, this β-casein-EGCG binding reduces the bioavailability of EGCG and causes a significant effect on the nutritional properties of the ultimate milk–tea formulation [2,172,173]. In contrast, β-casein–catechin binding imparts a pleasant taste to milk–tea by reducing the astringency taste of tea polyphenols [35,168,174,175]. In addition to β-casein, α-casein also interacts with EGCG through hydrogen bonding (Glu 85, Glu 132, Gln 93, and Asn 89), hydrophobic interactions (Pro 88, Pro 128, Ala 131, and Val 127) and van der Waals interactions (Glu 92, Val 91, Ser 130, and Ile 86) [172].

Casein–theaflavin interactions also showed a reduction in astringency in milk–tea. Brown and Wright (1963) [176] identified the reduction in astringency in tea caused by theaflavins with the addition of milk, due to interactions between polyphenols and proteins. The study was conducted using a 1:1 mixture of 3.5% tea solids infusion and a 20% solution of skim milk powder. The results identified the formation of soluble casein–polyphenol complexes (at 1:1 milk–tea ratio) with α- and β-caseins through hydrogen bonding. However, whey protein (β-Lg) was not involved in producing protein–phenol interactions at 70 °C and pH 6.25 (with 1:1 milk: tea ratio) when casein is present. In contrast, β-Lg either formed soluble or insoluble complexes with phenol in the absence of casein, depending on the tea concentration. For instance, β-Lg-phenol complexation occurred in a 1:1 protein: tea mixture prepared with 3.5% tea solid infusion and 0.3% of β-Lg solution at 70 °C (pH 6.45), whereas α-La complexation occurred in a 7:1 protein: tea solution with 0.05% α-La and 3.5% tea solid infusion. However, when the protein: tea proportion was beyond 7:1, the insoluble α-Lg-phenol complexation started to appear.

Mao et al. (2021) [37] reported that the color of liquid milk–tea is closely linked to the tea pigments and protein–polyphenol interactions. The hydrophobic and hydrophilic tea catechin, theaflavins (golden yellow), thearubigins (orange brown), and theabrownins (red brown/dark brown) interact with Pro-rich proteins (β-casein) to form more stable, insoluble protein conjugates, altering the original brown color of tea. Additionally, the color of milk–tea is influenced by the concentration of polyphenols. A low concentration of theaflavins and theabrownins results in an ivory or pink color, while an increase in theabrownins and theaflavins produces a reddish yellow or beige color in milk–tea. The lighter color of milk–tea with a low phenol concentration is due to the masking of all available proteins around phenol, while the darker color of milk–tea with a high phenol concentration is due to the remaining excess amount of phenol after protein masking [164].

**Figure 8:**
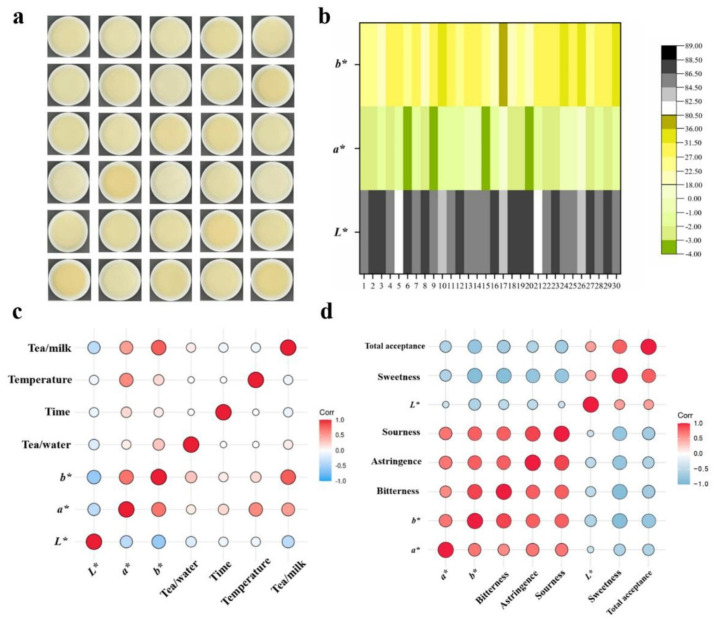
Milk–tea color alters with processing the conditions. (**a**) Thirty milk–tea samples (first above formulation-1, below right formulation-30); (**b**) milk–tea colors associated with various processing conditions and recipes; (**c**) effect of the processing conditions on milk–tea colors; and (**d**) milk–tea taste and color correlation [9]. Copyright 2023, Elsevier.

The color of tea can be influenced by protein–polyphenol interactions, which are affected by the type of tea, milk–tea ratio, and brewing conditions. The study conducted by Yang et al. (2022) [9] compared how the liquid milk–tea flavor varies with the tea type using six different teas, such as large leaf yellow tea, green tea, black tea, oolong tea, dark tea, and flower tea. Different teas were prepared by brewing 1 g of tea in 50 mL of water at 80 °C for 20 min and mixed with a similar proportion of milk and tea (1:1). The same study secondly evaluated the flavor and color of large leaf yellow milk–tea by preparing 30 different samples in which the brewing conditions, such as time (4, 12, 20, 28, and 36 min), temperature (60, 70, 80, 90, and 100 °C), tea:water (1:30, 1:40, 1:50, 1:60, and 1:70 g/mL), and tea:milk ratio (1:1, 1:2, 1:4, 2:1, and 4:1 mL/mL) were selected using a Box–Bhenken Design (BBD). The color of large-leaf yellow milk–tea prepared with various processing parameters observed the colors of dark-yellow, yellow, light-yellow, and ivory for the chromatic parameters ranging from 88.33 to 81, from 0 to (−3.67), and from 35.33 to 18.33 for L*, a*, and b*, respectively, as shown in Figure 8. These significant color changes were mainly attributed to the brewing temperature and tea–milk ratio. The tea:milk ratio and brewing temperature were significantly correlated with b* (29.33 to 31.33) values, respectively, where b* values correspond to the yellowness of the milk–tea. The milk–tea was darker when the tea:milk ratio was increased from 1:1 to 4:1 mL/mL and the brewing temperature was increased from 60 to 100 °C. This results from protein–phenol interactions filling up all the protein binding sites available, and the remaining excess polyphenols provide the medium a deeper hue.

The same study further investigated the correlation of leaf yellow milk–tea taste with the same brewing conditions, such as time (4, 12, 20, 28, and 36 min), temperature (60, 70, 80, 90, and 100 °C), tea:water (1:30, 1:40, 1:50, 1:60, and 1:70 g/mL), and tea:milk ratio (1:1, 1:2, 1:4, 2:1, and 4:1 mL/mL), using a Pearson correlation analysis. The L* and a* values, which indicate the lightness and degree of redness of milk–tea, respectively, were found to have a negative correlation with the bitterness (r = −0.543 and r = 0.639) and astringency (r = −0.484 and r = 0.688) of the milk–tea at a significance level of *p* < 0.01. The milk–tea bitterness and astringency both had strong positive correlations with the b* value (r = 0.856 and r = 0.838, respectively) at *p* < 0.01. The increased bitterness was associated with an increased tea proportion (tea:milk ratio, 2:1 and 4:1 mL/mL) and brewing temperatures (80, 90, and 100 °C), due to increases in bitter-related substances, including theaflavins and catechin (Prodelphinidin B4). An increase in the bitter compounds compared to proteins provides more unbound bitter compounds, and thereby, increases the bitterness. On the contrary, the sweetness of the milk–tea was decreased with the increase in the tea:milk ratio (1:1 and 1:2) and temperature (60 and 70 °C). This is mostly caused by the masking effect of milk sweetness by the increased bitterness of tea. However, the overall acceptance of large-leaf yellow milk–tea was maximized when the brewing temperature, brewing time, tea:water ratio, and tea:milk ratio were 89.8 °C, 27.04 min, 1:39.2 g/mL, and 1:2 mL/mL, respectively.

**Figure 9:**
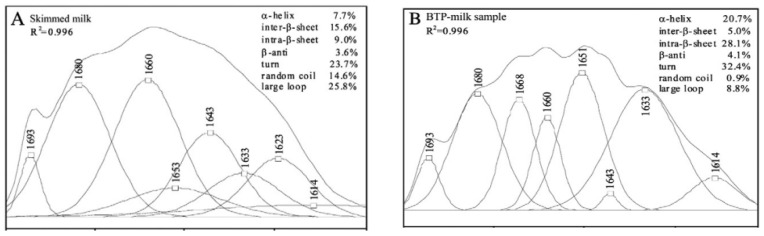
Milk protein secondary structure changes with and without polyphenols. The figure illustrates the enhancement in the second derivative resolution and the curve-fitted amide I region (1700–1600 cm^−1^) for skimmed milk (**A**) and black tea polyphenols (BTPs)–milk (**B**) analyzed by FTIR by [38]. Copyright 2023, Elsevier.

Zhang et al. (2014) [177] studied the association behavior of phenolic compounds and major whey proteins (β-Lg and α-La) using Fourier transform infrared spectroscopy (FTIR), fluorescence spectrophotometry, and circular dichroism (CD). The study identified the modified whey protein configurations with the presence of phenols due to a significant reduction in their α-helix in both β-Lg and α-La structures. The reduction in α-helix structures was caused by partial protein unfolding and hydrogen bonding between whey–phenol structures, ultimately attributed to lowering the stability of the final milk–tea formulation. However, these results were not aligned with the study conducted by Ye et al., (2013) [38]. Ye et al. (2013) [38] studied the influence of tea polyphenols–protein interactions on the secondary structure of the protein by means of FTIR in reconstituted milk–tea samples prepared with 40% (*v*/*v*) of skimmed milk and 60% (*v*/*v*) of a tea infusion brewed at 0.033 g/mL tea at 100 °C for 3 min. The FTIR spectra showed a significant increment in α-helix and intra-β-sheet from 7.7% to 20.7% and from 9.0% to 28.1%, respectively, in black tea samples, as shown in Figure 9. The study suggested that this α-helix and intra-β-sheet increment in protein (both casein and whey) was associated with the interaction between tea polyphenols and Pro residues of proteins. These interactions are driven by the strong affinity of phenols for the Pro residues on proteins. The protein–phenol interactions interrupt the random coil, large loop, and intra-β-sheet like irregular structures of proteins and release the protein segments to the medium. The released protein structures are thereby partially folded into α-helix and intra-β-sheet through hydrogen bonding. This causes an increase in the amount of α-helix and intra-β-sheet after protein–phenol interactions.

**Figure 10:**
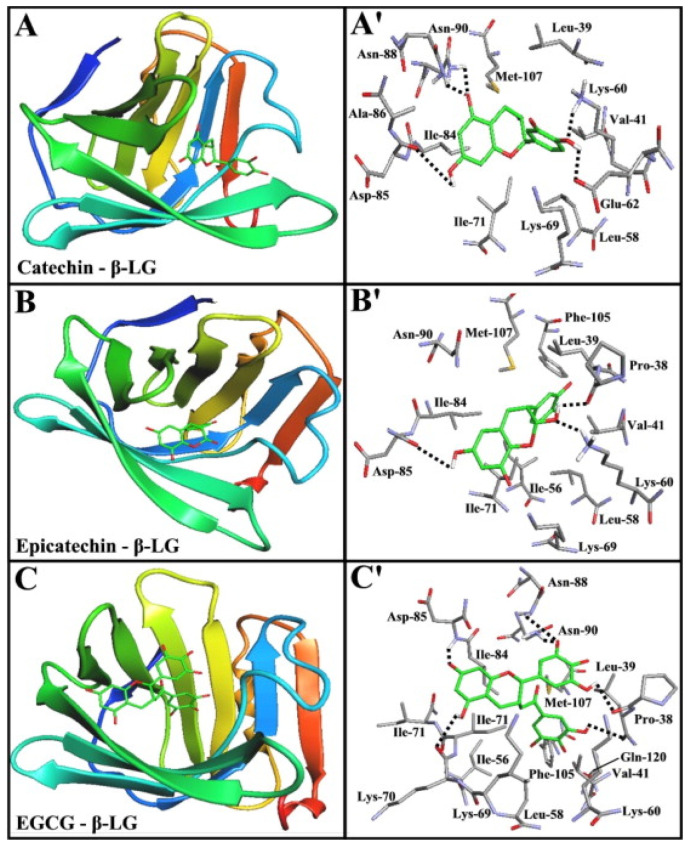
The docking position of β-Lg that forms the β-Lg-tea polyphenol interaction is shown in the figure. The red color indicates the residue of interest, while the green color shows the tea polyphenols. Panels (**A**–**C**) depict 3D diagrams of different polyphenol interactions with β-Lg, while panels (**A′**–**C′**) show 2D representations of the same polyphenol interactions with β-Lg. Panel (**A**): the binding positions of β-Lg-catechin, panel (**B**): the binding sites of β-Lg-epicatechin, and panel (**C**): the binding sites of β-Lg-EGCG [178]. Copyright 2023, Elsevier.

Kanakis et al. (2011) [178] identified the binding sites of β-Lg with catechin, epicatechin, and EGCG, as shown in Figure 10. The study identified that the amino acid residues Ala 86, Asn 88, Asn 90, Asp 85, Glu 62, Ile 71, Ile 84, Leu 39, Leu 58, Lys 60, Lys 69, Met 107, and Val 41 were involved in catechin-β-Lg interactions, while Asn 90, Asp 85, Ile 56, Ile 71, Ile 84, Leu 39, Leu 58, Lys 60, Lys 69, Met 107, Phe 105, Pro 38, and Val 41 were involved with epicatechin-β-Lg interactions in the medium. Moreover, Asn 88, Asn 90, Asp 85, Gln 120, Ile 56, Ile 71, Ile 84, Leu 39, Leu 58, Lys 60, Lys 69, Lys 70, Met 107, Phe 105, Pro 38, and Val 41 of β-Lg were associated with EGCG-β-Lg interactions. The study suggests that hydrogen bonding is involved in these interactions with catechin-β-Lg, epicatechin-β-Lg, and EGCG-β-Lg. However, the binding sites involved with catechin-β-Lg interactions observed by Kanakis et al. (2011) [178] did not align with the findings of Al-Shabib et al. (2020) [179]. Al-Shabib et al. (2020) [179] reported the amino acid residues involved in catechin-β-Lg binding (at pH 7.0 ± 2.0) were Pro 38, Leu 39, Val 141, Val 143, Ile 56, Leu 58, Ile 71, Ile 84, Val 192, Phe 105, Met 107, Lys 60, Glu 62, Lys 69, and Asn 90. The binding sites in this study differ from those in the earlier study conducted by Kanakis et al. (2011) [178] because they involve hydrophobic interactions instead of hydrogen bonding to stabilize the catechin-β-Lg complex. Therefore, the interactions between proteins and phenolic compounds, such as catechin, epicatechin, and EGCG, in liquid milk–tea involve hydrogen and hydrophobic interactions. However, there is not enough literature available on the interactions between protein and polyphenols at high solid concentrations in milk-containing mixtures.

Whey protein denaturation promotes the formation of a stable three-dimensional network that traps phenolic compounds during drying. After denaturation, a significant number of polyphenols bind to the hydrophobic core of whey proteins, particularly β-Lg. This binding occurs at three specific locations on β-Lg: the calyx (central cavity), surface cleft, and monomer/monomer interface. By binding to whey proteins, the polyphenol molecules are effectively shielded, preventing them from being exposed to the external environment. However, compared to casein, whey proteins have superior abilities to encapsulate phenols due to their efficient emulsification, film-forming, and gel-forming properties [180,181]. As a result, the encapsulation of polyphenols enhances their stability during storage, thereby improving the shelf life of milk–tea powder. Furthermore, encapsulating polyphenols helps to mask the unpleasant bitterness and astringent taste associated with phenols when consumed. The additional protection provided by proteins ensures the increased bioavailability of the final milk–tea powder [182].

### 4.4. Carbohydrate–Phenol Interactions

Carbohydrate–polyphenol interactions in liquid formulations occur either through covalent or non-covalent (hydrogen and hydrophobic) interactions [183]. The hydroxyl group of polyphenols in tea and oxygen atoms of the glycosidic linkage of the polysaccharide produce hydrogen bonds [184]. For instance, tannins (which are polymeric tea polyphenols) initiate extensive hydrogen interactions with carbohydrates due to the availability of high amounts of hydroxyl groups (OH) and higher hydrophobicity. Hydrophobic interactions occur between the aromatic rings of polyphenols and the hydrophobic sites of the carbohydrate. However, the carbohydrate–polyphenol interactions in liquid formulations depend on various factors, such as the molecular weight and hydrophobicity of polyphenols as well as the carbohydrate structure. These carbohydrate–polyphenol interactions do not impact the nutritional release of glucose in vivo [185]. The specific interactions between maltodextrin/lactose and tea polyphenols in liquid and concentrated milk formulas have not been studied yet.

The structure of maltodextrin enhances its ability to encapsulate polyphenols during drying. Specifically, the C1 and C4 carbons of maltodextrin are particularly responsive to conformational changes when polyphenols are present. The molecular size of the polyphenol determines whether the maltodextrin confirmation changes from a flexible coil to a helix shape. Hydrophobic interactions essentially involve in the interaction between maltodextrin and polyphenols under solid-state conditions. However, different dextrose equivalent (DE) values affect the intensity of the hydrophobic interaction between the core materials. Decreasing the DE value of maltodextrin (from 30 to 10) increases the encapsulation efficiency due to the containment of different cavity sizes with varying hydrolysis [186,187]. Using low DE maltodextrin in the spray drying process of milk–tea can improve the encapsulation efficiency of polyphenols, thereby maintaining the bioavailability of phenols. Lactose is also involved in the encapsulation of polyphenols due to its ability to form a large extent of amorphous structure, which easily traps the polyphenol. However, the presence of maltodextrin reduces the efficiency of lactose in encapsulation [188].

### 4.5. Protein–Carbohydrate–Polyphenol Interactions

Protein–carbohydrate–polyphenol interactions affect the stability of the final product. Wang et al. (2022) [27] studied the properties of whey–maltodextrin–polyphenol complexes in liquid formulations in terms of the particle size. For this, the whey–maltodextrin conjugates were prepared first by dissolving 1:1 powdered whey–maltodextrin conjugates and ultra-pure water to a concentration of 10 mg/mL and then combining 10 mg/mL of phenolic solution into the whey–maltodextrin conjugate solution at a final concentration of 0.3 mg/mL. The study observed that non-covalent interactions formed the whey–maltodextrin conjugate and whey–maltodextrin–polyphenol conjugates. Whey–maltodextrin–polyphenol conjugates showed a relatively lower particle size (at 56 nm) compared to whey–polyphenol complexes (165 nm) due to the conjugation of whey with maltodextrin prevents hydrophobic polymerization between proteins. The higher emulsion stability is due to lower whey–whey interactions. However, the lack of a significant difference between whey–maltodextrin and whey–maltodextrin–polyphenol suggested that non-covalent interactions had little or no effect on the increase in the particle size. Therefore, reducing the particle size prevents coalescing and phase separation, ultimately increasing the stability of the product. However, there is currently no research on the interactions between protein, carbohydrates, and polyphenols in concentrated milk-containing formulas.

During drying, heat-sensitive polyphenols are self-encapsulated with carbohydrates and/or proteins through various interactions, such as van der Waals interactions, hydrogen bonding, hydrophilic/hydrophobic interactions, or static interactions, due to quick heat exposure at high temperatures [189,190]. When atomizing the intensively homogenized phenolic compounds (core ingredient) and protein and/or carbohydrate solution (carrier solution) through a heated spray drying chamber, the phenolic compounds are wrapped by proteins and/or carbohydrates, while the water evaporates quickly from the particles [191]. This process of encapsulating tea polyphenols with carbohydrates and proteins enhances the heat stability of the polyphenols and preserves their bioavailability in milk–tea powder.

To deepen the understanding of protein–carbohydrate–polyphenol interactions, it is crucial to study how polyphenols modulate the Maillard reaction. Polyphenols impact the Maillard reaction by interacting with proteins and carbohydrates, influencing both its kinetics and the nature of its products. Han et al. (2024) [192] stowed the multifaceted role of polyphenols in this process, revealing that polyphenols, such as flavan-3-ols, hydroxycinnamic acids, flavonoids, and tannins, can inhibit the Maillard reaction at early stages by forming adducts with amino or thiol groups. While polyphenols can also react with sugars to form conjugates, the ability of specific polyphenols, like flavonoids or gallic acid, to do so remains still unclear. Furthermore, polyphenols can reduce the formation of heterocyclic aromatic amines and acrylamides, which are potential carcinogens and neurotoxins resulting from the Maillard reaction. For instance, in ultra-high-temperature (UHT) processed milk, phenolic acids, such as catechin and genistein, significantly inhibit the production of 2-acetyl-1-pyrroline, a Maillard reaction product. Non-flavonoid phenolics like hydroxytyrosol also inhibit the formation of off-flavor compounds, like methional and 2-acetyl-3-thiazoline. The study suggests that phenolic rings may be oxidized to quinones, which then react with lysine side chains and other amino groups to produce iminoquinone and iminophenol via Schiff base formation. Polyphenols also react with Maillard products and form low-molecular-weight products, such as polyphenol–α-dicarbonyl adducts, polyphenol–Strecker aldehyde adducts, and polyphenol–sugar addition products. Overall, the presence of polyphenols with proteins and carbohydrates together is beneficial as they modulate the Maillard reaction, potentially reducing harmful compounds and undesirable flavors while also contributing to the formation of novel, low-molecular-weight products. However, there is limited literature available on protein–carbohydrate–polyphenol interactions in both liquid and dried states, in addition to carbohydrate–polyphenol interactions.

The interactions among proteins, carbohydrates, and polyphenols are further influenced by their ratios. Table 2 summarizes how different ratios of these components affect their interactions and the resulting functionality of milk-containing formulations.

## 5. How Is the Overall Quality of Milk–Tea Affected by the Molecular Interactions of Its Composition?

One of the most appealing aspects of milk–tea is that milk is considered a complete food, while tea adds bioavailability to sustain the food. The overall quality of milk–tea, including yield, taste, color, aroma, stability, emulsifying ability, bioavailability, nutritional quality, allergenicity, shelf life, and solubility, is significantly influenced by component interactions as summarized in Table 3, during processing.

**Figure 11:**
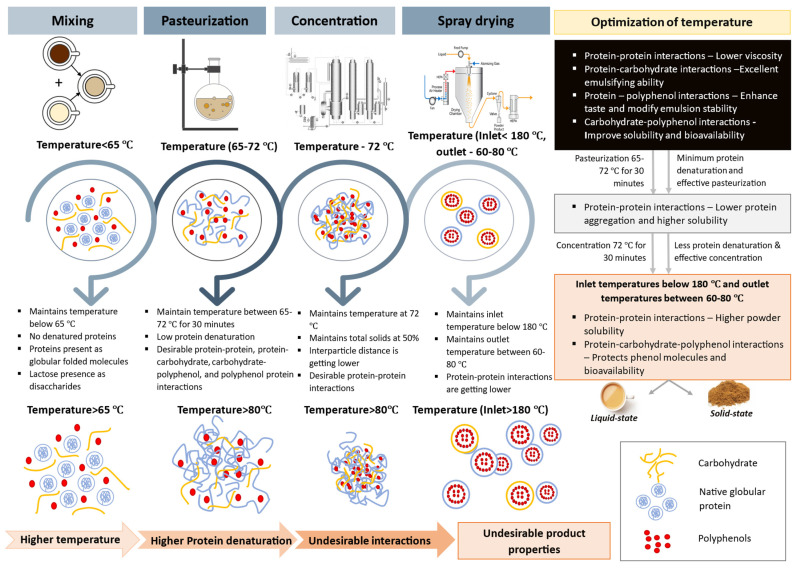
Figure illustrates the distribution of proteins, carbohydrates, and polyphenols, and the formation of interactions that occur during the mixing, pasteurization, concentration, and spray drying processes of milk–tea powder processing at both lower and higher temperatures. It further depicts the associated interactions between protein–protein, protein–carbohydrates, protein–polyphenol, carbohydrates–polyphenol, and protein–carbohydrates–polyphenol.

Whey proteins, in particular, have a pronounced effect on the quality, especially during pasteurization, compared to concentration and spray drying [207]. Protein–protein interactions influence functional properties like viscosity in liquid milk–tea and affect hydration, solubility, dispersibility, and wettability in the final powders [203,208]. Incorporating maltodextrin into milk to partially replace lactose can help to mitigate whey protein aggregation, thereby improving the final product solubility. Maltodextrin achieves this by forming hydrogen bonds that stabilize protein structures and preserve their native α-helix content, thereby preventing undesirable conformational changes [87]. Additionally, the partial substitution of maltodextrin influences the Maillard reaction, contributing to a desirable yellow-brown color in milk–tea and enhancing sensory attributes, such as flavor and aroma. The Maillard reaction also produces antioxidative compounds that help to reduce oxidative damage and extend the product’s shelf life [135,136,209]. By limiting excessive Maillard reactions between lactose and proteins, maltodextrin prevents undesirable changes and imparts favorable properties to the final product [210,211]. Additionally, maltodextrin enhances protein binding and emulsifying properties, which benefits the final product’s quality [149]. Proteins that form a protective matrix around polyphenols can enhance sensory attributes, such as taste, color, aroma, and texture, in milk–tea by mitigating the strong effects of polyphenols [1,9]. Although this complexation may decrease the bioavailability of tea polyphenols, it can simultaneously increase the bioavailability of milk components [212]. Achieving these quality improvements often relies on the interactions among proteins, carbohydrates, and polyphenols, as well as specific processing conditions as illustrated in Figure 11. The optimal quality is typically achieved through low-heat applications and the careful management of these interactions, particularly concerning whey protein denaturation temperatures.

**Figure 12:**
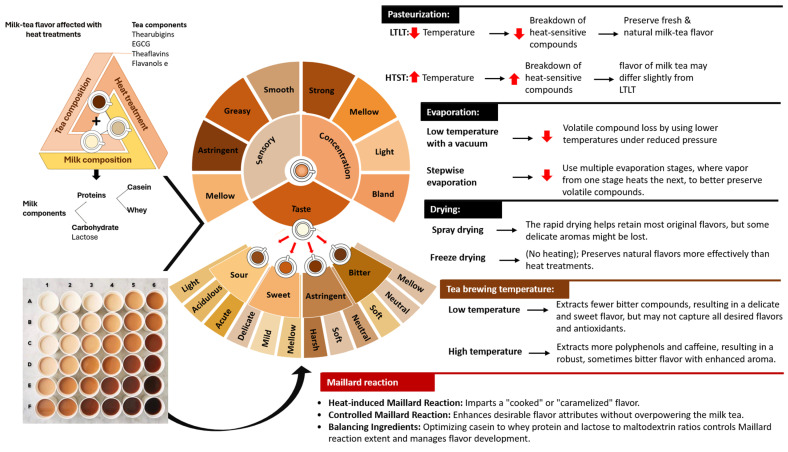
Impact of various heat treatments on milk–tea flavor development. This figure illustrates the effect of different heat treatments on the flavor profile of milk–tea mixes.

Flavor plays a significant role among all quality attributes and can be easily affected by composition and processing conditions. Therefore, Figure 12 summarizes how the flavor of milk–tea formulated with black tea is influenced by its composition and various processing stages.

Polyphenols play a crucial role in defining the flavor profile of both tea and milk–tea. In black tea, catechins are converted during fermentation into theaflavins, thearubigins, and theabrownins, which contribute to the tea’s distinctive bitter and astringent taste. This robust flavor profile, coupled with a high dissolution rate, makes black tea a popular choice for milk–tea formulations [37,213,214]. The taste of milk–tea is complex and includes four main sensations, bitter, sour, sweet, and astringent, all of which are significantly influenced by the type of tea used [9].

The addition of milk to tea reduces bitterness through interactions between polyphenols and milk components. Hydrogen bonding between proteins and polyphenols decreases the astringency of tea polyphenols, contributing to a more pleasant taste in milk–tea [35,168,174,175]. Polysaccharides also play a role by masking unpleasant flavors through polar, hydrogen, or dipole–dipole interactions, which can alter the flavor intensity and sometimes render it weaker or tasteless [75]. While these interactions reduce bitterness, milk increases the sweetness of the tea.

Furthermore, the taste of milk–tea is influenced by several key factors, including the type of tea used, brewing temperature, tea-to-milk ratio, and processing temperature. As the tea concentration decreases (at lower tea-to-milk ratios), the flavor profile transitions from strong to mellow, light, and eventually bland. The optimal brewing conditions are identified as 89.8 °C for 27.04 min with a tea-to-water ratio of 1:39.2, alongside an ideal tea-to-milk ratio of 1:2, which enhances the overall consumer acceptance. The type of tea, whether black or green, also affects the flavor differently [9,37]. Additionally, high temperatures during processing stages, such as pasteurization, evaporation, and spray drying, can significantly alter the flavor and volatile compounds of milk–tea. These thermal processes can lead to the formation of Maillard reaction products, which can influence the natural flavor of the final product. While some Maillard products can enhance the flavor by adding complexity and depth, others may result in undesirable off-flavors or reduce the intensity of the original taste profile [215,216].

## 6. Implications that Arise during the Current Milk–Tea Manufacturing Process

The production process of milk–tea involves several factors that significantly impact the overall quality of the final product. Key factors including, ingredient composition and processing conditions, are summarized in Table 4. Ingredient consistency, particularly in tea and milk, can lead to notable differences in flavor, texture, and the overall sensory experience. Additionally, the processing conditions, such as brewing temperatures, mixing ratios, blending methods, and heat treatments, play crucial roles in determining the final attributes of milk–tea. 

## 7. Future Trends

Previous research has primarily focused on interactions in the liquid state, such as protein–protein, protein–polyphenol, and protein–carbohydrate interactions. However, there is a lack of research on interactions between maltodextrin, lactose, and tea polyphenols during the pasteurization and evaporation processes. It is important to further explore lactose–lactose, lactose–maltodextrin, maltodextrin–maltodextrin, lactose–polyphenol, and maltodextrin–polyphenol interactions, as preheating and evaporation can significantly alter component structures. Additionally, there has been a lack of studies on protein–polyphenol interactions during concentration, and this area also requires further investigation.

The concentration of various components in milk–tea can impact interaction pathways differently. The optimal ratios of components such as lactose, maltodextrin, casein, and whey can significantly influence the interactions. These interactions enhance the physical, structural, nutritional, and functional properties of milk-containing formulations. However, these ratios have not been studied in the context of milk–tea formulations. Research on how different concentrations of casein, whey, lactose, maltodextrin, and tea behave during preheating, concentration, and spray drying, and how modifying their interactions affects their properties in milk–tea, is yet to be conducted.

Many of the negative qualities in milk are caused by heat-induced protein interactions especially during pasteurization. Using non-thermal techniques, such as ultrasound, high-pressure processing, and pulsed electric field, instead of preheating, which are cost-effective, can effectively ensure microbial safety while preserving the natural protein structures. Additionally, ultrasound can be combined with pressure during pasteurization [227,228]. Reverse osmosis (RO) is also reported as a cost-effective technique that does not involve heat application for concentration [229]. While freeze-drying is a great method for drying heat-sensitive compounds like polyphenols, it is expensive for use in industry [226]. Therefore, using novel spray drying techniques, such as polar drying, which utilizes moderate drying conditions, may preserve the natural properties of milk components and achieve the desired characteristics of milk–tea powder. Hence, it is necessary to concentrate on the investigation of non-thermal processing and novel spray drying techniques in the production of milk–tea powder [98,230].

## 8. Conclusions

Optimizing the interactions between protein, carbohydrate, and polyphenol components during pasteurization, concentration, and spray drying can enhance the properties of milk–tea powder produced through the wet mixing method. Pasteurization leads to protein–protein interactions, resulting in a larger particle size and higher viscosity. Concentration-induced protein–protein interactions further enhance viscosity. Minimizing protein–protein interactions during spray drying is crucial for increasing powder solubility. Protein–carbohydrate interactions mainly occur through the Maillard reaction. This reaction, which occurs at each step, affects the taste and heat stability of the powder while reducing its nutritional value and digestibility. During dehydration, protein–carbohydrate interactions preserve the protein structure and minimize dehydration-induced protein damage. Pasteurization-induced protein–polyphenol complexes not only improve the flavor of milk–tea but also alter its stability and color. Additionally, the interactions between protein and polyphenols during drying enhance their bioavailability and stability during storage, while also preventing the unpleasant bitterness or astringent taste associated with phenols. Protein–carbohydrate–polyphenol interactions during drying affect the stability of the final product. This knowledge enables a better control of undesirable interactions and the improvement in desirable interactions, ultimately leading to the development of a more rational milk–tea powder formulation.

In the current food manufacturing industry, optimizing the interactions between proteins, carbohydrates, and polyphenols is vital for enhancing the quality of milk–tea products. Balancing the ratios of casein to whey protein is essential for achieving the desired functionality and stability in milk–tea powder, which affects the product’s texture and mouthfeel. Managing lactose to maltodextrin ratios is critical for mitigating protein aggregation and controlling Maillard reaction effects, thereby tailoring the sensory attributes, such as flavor and color. The incorporation of maltodextrin as a partial substitute for lactose not only reduces protein aggregation but also improves solubility and sensory appeal through controlled Maillard reactions. Further, investigating the effects of different tea sources can increase the bioavailability and sensory properties of milk–tea. Conducting consumer sensory studies to gather feedback on different formulations can further refine product development. Additionally, employing non-thermal processing techniques, such as ultrasound or high-pressure processing, can preserve natural protein structures and bioactive compounds, ensuring microbial safety while enhancing the functional properties of the milk–tea powder.

## Figures and Tables

**Table 1:** The summary of the factors affecting the Maillard reaction.

Factor	Method and Conditions	Effect on the Maillard Reaction	Reference
Heating method	Wet heating: Uses high temperatures ~90 °C for 2–96 h	High heat alters the protein structure inevitably and affects protein functionality, including foaming and emulsifying properties.	[138]
	Dry heating: Uses a temperature in the range of 0–80 °C for a longer time	Protein denaturation and aggregation rarely occur due to the use of mild heat treatment, resulting in improved stability during long-term storage.	[139]
Temperature	Occurs above 35 °C	At a temperature of 35 °C, the Maillard reaction occurs slowly and accelerates at or above 55 °C. The structure of proteins changes at high temperatures due to denaturation, aggregation, and precipitation, which reduces the number of amino groups that can participate in the reaction.	[140,141]
pH	When pH > 7	The earlier stage of the Maillard reaction progresses more quickly.	[141]
	When pH is up to 10	The polysaccharides in open-chain form, which are usually favored at higher pH levels up to 9–10, exhibit the maximum reactivity.	
Water activity	In the range of 0.60–0.85	The Maillard reaction demonstrates the highest possible reaction rate.	[142]
Water content	In the range of 30–75%	Increasing the water content accelerates the Maillard reaction.	
Carbohydrate type	Monosaccharaides/disaccharides/ oligosaccharides /polysaccharides	The degree of the Maillard reaction can be limited by substituting polysaccharides for the reducing sugars because they have fewer reducing groups and a higher molecular weight.	[143]

**Table 2:** The summary of the literature found for physiochemical and functional changes in liquid, semi-solid, and solid-state proteins, carbohydrates, and polyphenols containing formulations with varying composition ratios.

Sample	Treatment Conditions	Type and Impact of Interactions	Functionality Modifications	References
Liquid milk formula that maintained a C:W ratio of proteins (5.5% total protein) at 40:60 with increasing α-La:β-Lg ratios at 0:1, 0:5, 1:3, 2:1, and 4:6	Stability at high temperature (140 °C and pH 6.6–6.9) and viscosity changes during HTST treatment were observed	Protein–protein interactions	Protein–protein interactions (whey–casein association) were decreased with increasing α-La:β-Lg ratios. This increased heat stability and showed a less extensive increase in particle size, viscosity, and covalent interactions between proteins after thermal applications	[107]
Liquid milk formula that maintained a C:W ratio of proteins (1.45% total protein) at 94:6, 90:20, 60:40, 40:60, 20:80, and 7:93	Forming properties of skim milk against heating at pH 6.6 were investigated	Columbic interactions (electrostatic interactions between electric charges)	Increased bubble diameter (d10) and higher foam density in the range of 0.15–0.16 g/cm^3^ were observed in the 60:40 and 20:80 samples. A C:W ratio of 20:80 and a pH of ≤6.7 exhibited attractive foaming properties	[193]
Liquid infant milk formula produced by maintaining the C:W ratio of proteins (15% *w*/*w* protein from 20% *w*/*w* TS) at 40:60, 50:50, and 60:40	Particle size, zeta potential, and viscosity of UHT pasteurized (100 °C for 30 min) and homogenized (at 55 °C with first- and second-stage pressures of 13.8 MPa and 3.5 MPa, respectively) wet mix of pH 6.8 was evaluated	Electrostatic repulsion	The particle size and viscosity did not significantly differ with different C:W ratios. The particle size of all samples was below 1 µm and reflected better emulsification properties for both casein and whey protein. The sample at 40:60 reported the highest net negative charge, and it decreased significantly with the increase in casein fractions. Sufficient electrostatic repulsion between droplets maintains wet mixes stable by preventing attractive interactions between droplets	[194]
Liquid infant milk formula produced by maintaining the L:M ratio of carbohydrates (59% *w*/*w*) at 100:0, 85:15, and 70:30. The formula contained 15% *w*/*w* of protein at C:W ratio of 40:60	Droplet size, zeta potential, and viscosity of the UHT pasteurized (100 °C for 30 min) and homogenized (at 55 °C with first- and second-stage pressure of 13.8 MPa and 3.5 MPa, respectively) wet mix of pH 6.8 was evaluated	Electrostatic repulsion	Droplet sizes were not significantly different with L:M ratios. The particle size of all samples was below 1 µm. The net negative charge was the greatest for the sample without maltodextrin, followed by L:M ratios of 85:15 and 70:30. All samples had sufficient electrostatic repulsion to prevent droplet attraction associations. There was an insignificant increase in the viscosity of samples with the increase in the maltodextrin concentration	[195]
Liquid whey–phenol solution prepared using WPI and EGCG by maintaining the WPI concentration at 5 mg/mL while varying the ECCG concentration to obtain WPI:EGCG ratios of 1:1, 1:0.5, 1:0.2, and 1:0.1	The solution was tested for its allergenic properties at two different pH values at 3.5 (acidic) and 7.0 (neutral) and temperature at 25 °C	Non-covalent interactions occurred as a consequence of whey protein’ secondary and tertiary structure modifications	The WPI-EGCG complexes at a molar ratio of 1:1 at both pH levels showed a lower IgE binding to β-Lg and BSA, which are two allergens in milk (but not to α-La). The complexation of EGCG causes the formation of hypoallergenic products and, therefore, effectively reduces the allergenicity of β-Lg and BSA	[196]
Concentrated infant milk formula produced by maintaining the C:W ratio of proteins (15% *w*/*w* protein from 20% *w*/*w* TS) at 40:60, 50:50, and 60:40	Particle size, zeta potential, and viscosity of evaporated (TS—50 ± 2%) wet mix at pH 6.8 was evaluated	Electrostatic repulsion	The volume mean diameter of all C:W ratios increased with evaporation due to the coalescence of emulsion droplets. The concentrated sample, which had a C:W ratio of 60:40, showed the largest particle size, followed by 50:50 and 40:60. The net negative charge was the highest for 40:60 and decreased with the increase in casein fractions. Sufficient electrostatic repulsion between droplets helped to keep the wet mixes stable. The viscosity of the samples decreased with the increase in casein content and was attributed to a higher whey protein denaturation	[194]
Concentrated infant milk formula produced by maintaining an L:M ratio of carbohydrates (59% *w*/*w*) at 100:0, 85:15, and 70:30. The formula contained 15% *w*/*w* of proteins at a C:W ratio of 40:60	Droplet size, zeta potential, and viscosity of the concentrated (TS 50 ± 2%, pH 6.8) infant formula using falling film evaporator was analyzed	Electrostatic repulsion	Although the droplet size of the samples was not different for L:M ratios, the concentration showed an increment. The net negative charge was the greatest for the sample without maltodextrin, followed by the samples bearing L:M ratios of 85:15 and 70:30. However, the negative charge decreased after evaporation. Sufficient electrostatic repulsion between droplets helped to keep the wet mixes stable. The apparent viscosity of the concentrate increased with the increase in the maltodextrin concentration	[195]
Concentrated milk powder formulas that maintained a C:W ratio of proteins (modulating protein contents 10, 14, and 18/100g) at 60:40, 40:60, and 80:20	Viscosity, bulk density, and particle size were observed in spray-dried (inlet temperature—185 °C, outlet temperature—90 °C, water evaporation rate—20 L/h) powders	Polymer–polymer interactions	Increasing the protein content and decreasing the whey protein to casein ratio were observed to increase viscosity during processing. At the C:W ratio of 80:20, particle size, viscosity, and bulk densities were higher than in the samples that had a C:W ratio of 40:60	[197]
Infant milk formula powder produced by maintaining a C:W ratio of proteins (15% *w*/*w*) at 40:60, 50:50, and 60:40	Particle size, particle morphology, water activity, color, bulk and surface composition, crystallinity, and solubility of spray-dried (inlet temperature—180 °C, outlet temperature—90 °C, rotational speed of rotary atomizer—21,500 rpm, feed temperature—55 °C) infant formulas were observed	Covalent disulphide bonds	The power bulk compositions (total protein, fat, carbohydrate, and ash) were not significantly different after spray drying at different C:W ratios. The volume mean diameter of the powders increased with the increase in the C:W ratios. A lower water activity was reported by the C:W ratio at 60:40. Glass transition temperatures (Tg), crystallinity, surface composition, color, and solubility of the powders were not significantly affected by the C:W ratios. The surface morphology of all freshly prepared powders was mostly smooth, spherical, and with little or no agglomerations. Whey–casein covalent disulfide bonds showed up to a little extent, and hence, showed some degree of aggregation	[194]
Infant milk formula powder produced by maintaining an L:M ratio of carbohydrates (59% *w*/*w*) at 100:0, 85:15, and 70:30. The formula contained 15% *w*/*w* of proteins at a C:W ratio of 40:60	Powder composition, particle size, water activity, glass transition temperature, crystanillity, surface morphology, surface composition, free fat, color, and solubility of spray-dried powder (inlet temperature—180 °C, outlet temperature—90 °C, atomizer pressure—0.3 MPa) were analyzed	Covalent disulphide bonds	The powder composition remained unchanged in contrast to the initial composition. The moisture content, crystallinity, and yellowness of the powders gradually decreased with the increase in the maltodextrin content. The particle size, water activity, solubility, and surface composition (proteins, carbohydrates, fats) did not significantly differ among the L:M ratios. The smallest particles were observed at the L:M ratios of 100:0 (50 µm), 85:15 (51 µm), and 70:30 (51.3 µm). Tg significantly increased with the increase in the maltodextrin concentration. The surface morphology of all freshly prepared powders was mostly smooth, spherical, and with little or no agglomerations. The presence of aggregations indicated the formation of covalent disulphide bonds	[195]
Casein–lactose model powder matrix prepared with a C:W ratio at 1:0, 1:1.5, 1.2, and 1:2.5	Color, protein aggregation, and protein structure changes were investigated in terms of the effect on spray-dried powder (inlet temperature—175 °C, outlet temperature—80 °C, peristaltic pump speed—11 mL/min)	Non covalent bonding (hydrogen and hydrophobic interactions)	Casein glycation was not dependent on the relative lactose amounts, and there was no difference in the browning index after spray drying. However, glycation resulted in larger molecules in the 1:1.5 C:L fortified powder, associated with α-casein and β-casein glycation sites. Both the 1:2 and 1:15 C:L ratios showed fluorescence of casein lower than 2.0 × 1010 M-1S-1, indicating the presence of non-covalent bonds between casein and lactose molecules. Upon glycation, new hydroxyl groups were introduced to the structure. The ratio of 1:1.5 reported the highest glycation. Glycation reduced the casein hydrophobicity, and particle distribution followed with casein aggregation	[198]

Abbreviations: C:W—casein: whey; HTST—high-temperature short-time; UHT—ultra-high temperature; L:M—lactose:maltodextrin; WPI—whey protein isolate; lgE—immunoglobulin E; C:L—casein: lactose.

**Table 3:** The summary of the literature found for physiochemical and functional property modifications with respect to protein, carbohydrate, and polyphenol interactions.

Powder Property	Associated Interaction	Correlation of Desired Powder Property with Associated Interaction	References
Flavor quality	Protein–protein interactions (whey–casein interactions)	Decrease the flavor quality with the increase in protein–protein interactions due to losing proteins with precipitation	[25]
	Polyphenol–protein–polysaccharides	Reduce the astringency taste of polyphenols with polysaccharides–flavonol–BSA interactions as BSA α-helical structures become curled irregular and, hence, precipitate. Therefore, polyphenol–protein–polysaccharides interactions thicken and mellow the taste of the medium	[199]
Color	Protein–polyphenol complexes (theaflavins, thearubigins, and EGCG–milk protein)	Modify interactions with tea polyphenols/pigments and proteins provide redness to the milk–tea where there was no visible red in tea before mixing in milk	[37]
	Whey–maltodextrin conjugates	Provide yellowish to dark brown color depend on the heating time due to Maillard conjugation	[200]
Antioxidant capacity	Protein–polyphenol interactions (β-Lg-EGCG)	Change the antioxidant capacity mainly due to structural changes in the β-Lg molecule because binding polyphenols to proteins changes its secondary structure with the increase in β-sheets and α-helix followed by the structure stabilization of proteins	[178]
	Protein–polyphenol interactions β-casein-EGCG complexes	Binding β-casein with EGCG reduces the antioxidant properties of EGCG due to effects on the electron donation ability of polyphenol by reducing its available free hydroxyl groups to oxidize	[2,201]
	Protein–polyphenol conjugates	Binding proteins with phenols increases the antioxidant ability of proteins in contrast to proteins alone	[21]
Nutritional availability	Protein–polyphenol complexes	Nutritional properties of proteins are reduced with protein–polyphenol complexes due to lowering the availability of amino acids	[202]
	Protein–carbohydrate interactions (through the Maillard reaction)	Modify the available lysine’s residence in proteins during the Maillard reaction, resulting in the lower availability of amino acids, and hence, a lower nutritional value	[126,129]
Foaming properties	Protein–polysaccharide complexes	Foaming properties obtained with protein–polysaccharide complexes are considerably higher than protein alone due to the increase in the stability of interfacial liquid film. These viscoelastic properties of protein–polysaccharide complexes entrap air to form stable foams in the system. Increase in the viscosity in the liquid film with these complexes in the mixture also increases the foam stability due to lowering the air diffusion, entrapped inside the foam	[66]
Solubility	Protein–protein and protein–carbohydrate interactions (casein–casein, casein–whey, and protein–lactose)	Casein–casein and casein–whey interactions are the main cause of the insolubility of powders. A greater insolubility is then promoted by protein–lactose during the Maillard reaction	[203]
	Whey–carbohydrate interactions	Partially glycosylated whey enhanced solubility and heat stability due to suppressing inter-molecular interactions, thereby resulting in resistance to denaturation and reduced surface hydrophobicity. The intermolecular interaction reduction is caused with unique glycosylation sites and lowering sulfhydryl sites	[204]
	Whey–maltodextrin conjugates	Significantly improved protein solubility of whey–maltodextrin conjugates are attributed to enhanced protein hydration. Furthermore, whey–maltodextrin conjugates provide a superior thermal stability to proteins in an added salt environment	[200]
	Protein–polyphenol complexes	The solubility of the protein is decreased due to the increase in the molecular weight of proteins by protein–polyphenol conjugation	[205]
Allergenicity	Protein–polyphenol complexes (β-Lg-catechin)	Binding β-Lg with tea catechin yielded a lower allergenicity due to shielding epitopes of β-Lg, which lower the binding capacity of IgE and IgG	[206]

Abbreviations: BSA—blood serum albumin; EGCG—epigallocatechin gallate, β-Lg—β-lactoglobulin; IgE—immunoglobulin E; IgG—immunoglobulin G.

**Table 4:** Summary of implications arising during milk–tea production and scientific explanation of their impact on quality attributes.

Milk–Tea Processing Stage	Implication	Scientific Explanation	Reference
Ingredient consistency	Difficulty of maintaining the composition of tea and milk	Variations in the levels of polyphenols and the degree of fermentation of catechins in tea leaves can lead to significant differences in the astringency, bitterness, and overall flavour profile of milk–tea. The composition of polyphenols in tea is highly dependent on the type of tea, tea growing conditions, and processing methods. Variations in milk composition, such as the presence of fat, being skimmed, or using fermented milk, affect the overall quality, richness, and texture of the final product. Milk fat composition is significantly influenced by short-chain fatty acids, notably butyric acid, which contributes to the characteristic flavour and creaminess of milk.	[213,217,218,219,220,221,222]
Processing conditions	Brewing temperatures of tea Mixing ratio of milk: tea Method of blending	Different brewing temperatures affect the extraction of polyphenols, catechins, and other compounds from tea leaves, impacting the flavour, aroma, and health benefits of the final milk–tea product. Higher temperatures typically extract more polyphenols, leading to a stronger, more astringent flavour, while lower temperatures extract fewer polyphenols, resulting in a milder taste. The ratio of milk to tea can influence the balance of flavors and the overall sensory experience of the milk–tea. A higher proportion of milk can mellow the astringency and bitterness of tea, while a lower proportion of milk preserves the robustness of the tea flavors. The optimal ratio depends on the desired sensory attributes of the final product. The method of combining milk and tea can significantly modulate the astringency of the final product. Adding milk to strong black tea is recommended due to its higher ability to mellow out tea tannins, resulting in less bitterness. This approach optimizes the interaction between milk proteins and tea polyphenols, effectively reducing astringency and enhancing flavour balance.	[35,76,223]
Heat treatment	Method and the temperature of pasteurization	The method and temperature of pasteurization can affect the quality and safety of milk–tea. Pasteurization is essential to eliminate pathogenic microorganisms and extend shelf life. However, excessive heat treatment can lead to undesirable changes in the sensory attributes and nutritional profile of the final product.	[224]
	Method of concentration	Evaporation reduces the final product quality due to prolonged heat exposure, negatively affecting color, taste, and nutritional value. Reverse osmosis (RO) has a lower energy consumption and less heat exposure, improving product properties, but is affected by membrane fouling and fat globule damage, which increases free fatty acids, challenging for whole milk.	[225]
	Method of drying	Drying method affects the total phenolic content. High temperatures in spray drying degrade phenolic compounds, reducing the total phenolic content. Freeze-drying at low temperatures preserves phenolic compounds, maintaining a higher total phenolic content.	[226]

## Data Availability

No new data were created or analyzed in this study. Data sharing is not applicable to this article.
